# *catena*-Poly[[di­chlorido­mercury(II)]-μ-1,4-bis­[2-(pyridin-4-yl)ethyn­yl]benzene-κ^2^ *N*:*N*′]

**DOI:** 10.1107/S1600536814010228

**Published:** 2014-05-10

**Authors:** Bin Wang, Ming Li, Ya-bo Xie

**Affiliations:** a State Key Laboratory Base of Eco-Chemical Engineering, College of Chemistry and Molecular Engineering, Qing Dao University of Science and Technology, Qingdao 266042, People’s Republic of China; b Department of Chemistry and Chemical Engineering, College of Environmental and Energy Engineering, Beijing University of Technology, Beijing 100124, People’s Republic of China

## Abstract

In the polymeric title compound, [HgCl_2_(C_20_H_12_N_2_)]_*n*_, the Hg^II^ atom is located on a twofold rotation axis and the benzene ring of the bidentate bridging 1,4-bis­[2-(pyridin-4-yl)ethyn­yl]benzene (*L*) ligand is located about a twofold rotation axis. The Hg^II^ atom is coordinated by two N atoms of two different *L* ligands and by two chloride ions in a distorted tetra­hedral geometry. The dihedral angle between the coordinating pyridine and the benzene ring is 12.8 (2)°. The result of the bridging is the formation of a zigzag chain running parallel to [102]. The chains pack with no specific inter­molecular inter­actions between them.

## Related literature   

For examples of 1,4-bis­[2-(pyridin-4-yl)ethyn­yl]benzene-containing polymers, see: Yamada *et al.* (2011 ▶). For examples of Hg-containing polymers, see: Xie & Wu (2007 ▶). For the synthesis of the ligand, see: Fasina *et al.* (2004 ▶).
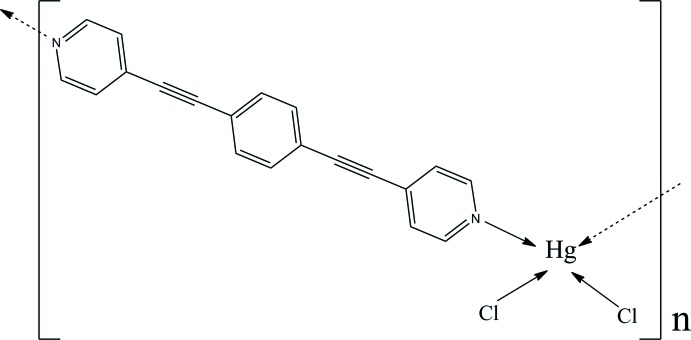


## Experimental   

### 

#### Crystal data  

[HgCl_2_(C_20_H_12_N_2_)]
*M* *_r_* = 551.81
Monoclinic, 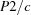

*a* = 12.285 (3) Å
*b* = 4.8482 (10) Å
*c* = 15.271 (3) Å
β = 98.00 (3)°
*V* = 900.7 (3) Å^3^
*Z* = 2
Mo *K*α radiation
μ = 8.85 mm^−1^
*T* = 173 K
0.18 × 0.16 × 0.16 mm

#### Data collection  

Bruker SMART 1000 CCD area-detector diffractometer
Absorption correction: multi-scan (*SADABS*; Sheldrick, 1995 ▶) *T*_min_ = 0.222, *T*_max_ = 0.243
4238 measured reflections
1585 independent reflections
1512 reflections with *I* > 2σ(*I*)
*R*_int_ = 0.033

#### Refinement  

*R*[*F* ^2^ > 2σ(*F* ^2^)] = 0.028
*wR*(*F* ^2^) = 0.070
*S* = 0.92
1585 reflections
114 parameters
H-atom parameters constrained
Δρ_max_ = 2.15 e Å^−3^
Δρ_min_ = −1.73 e Å^−3^

### 

Data collection: *SMART* (Bruker, 1998 ▶); cell refinement: *SAINT* (Bruker, 1998 ▶); data reduction: *SAINT*; program(s) used to solve structure: *SHELXS97* (Sheldrick, 2008 ▶); program(s) used to refine structure: *SHELXL97* (Sheldrick, 2008 ▶); molecular graphics: *SHELXTL* (Sheldrick, 2008 ▶); software used to prepare material for publication: *SHELXTL*.

## Supplementary Material

Crystal structure: contains datablock(s) 1, I. DOI: 10.1107/S1600536814010228/tk5312sup1.cif

Structure factors: contains datablock(s) I. DOI: 10.1107/S1600536814010228/tk5312Isup2.hkl

Supporting information file. DOI: 10.1107/S1600536814010228/tk5312Isup3.cdx

CCDC reference: 1001220

Additional supporting information:  crystallographic information; 3D view; checkCIF report

## Figures and Tables

**Table 1:** Selected bond lengths (Å)

Hg1—Cl1^i^	2.3719 (12)
Hg1—Cl1	2.3719 (12)
Hg1—N1^i^	2.412 (3)
Hg1—N1	2.412 (3)

Symmetry code: (i) 
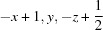
.

**Fig. 1.**
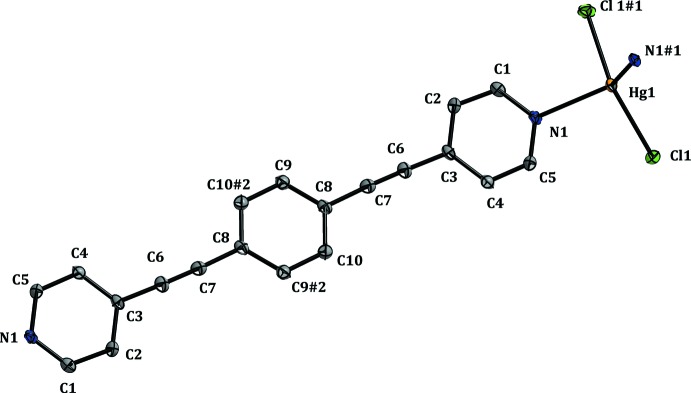
The coordination mode of the title complex, with displacement ellipsoids drawn at the 50% probability level. All H atoms have been omitted for clarity. [Symmetry codes: (#1) -x+1, y, -z+1/2; (#2) -x, -y+3, z+1.]

**Fig. 2.**
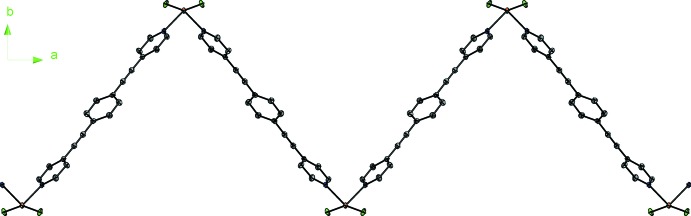
The zigzag chain of the complex. View down the c axis, with displacement ellipsoids drawn at the 50% probability level. All hydrogen atoms are omitted for clarity.

## supplementary crystallographic information

### 2. Structural commentary

Recently, a large number of coordination polymers assembled from pyridyl-based ligands have been extensively investigated. Most of these coordination polymers are constructed from 4,4'-bi­pyridyl but other examples of bridging ligands are known, such as with 1,4-bis­(pyridin-4-ylethynyl)benzene (Yamada *et al.*, 2011). Mercury coordination polymers are known (Xie *et al.*, 2007)

In this work, an linear pyridyl-based ligand, 1,4-bis­(pyridin-4-ylethynyl)benzene, was employed to react with HgCl_2_ to afford the title complex, [Hg(C_20_H_12_N_2)_Cl_2]_]_n_ (I). In I, the Hg(II) center is coordinated by two N atoms of two different 1,4-bis­(pyridin-4-ylethynyl)benzene ligands and two chloride ions in a distorted tetra­hedral geometry (Fig. 1). The Hg(II) centers are linked by 1,4-bis­(pyridin-4-ylethynyl)benzene ligands to form a one-dimensional zigzag chain and the chain is parallel to [102] (Fig. 2). The dihedral angles between coordinated pyridine rings and benzene ring are *ca.* 12.8 (2)°.

### 5. Synthesis and crystallization

The ligand 1,4-bis­(pyridin-4-ylethynyl)benzene (bpyb) was synthesized from the reaction between 4-(prop-1-yn-1-yl)pyridine and 1,4-di­iodo­benzene following the reported procedure (Fasina *et al.*, 2004). A methanol (3 ml) solution of HgCl_2_ (0.1 mmol, 27 mg) was layered upon a chloro­form solution (3 ml) of bpyp (0.2 mmol, 56 mg). After three days, colourless crystals of the title complex suitable for X-ray analysis were obtained.

### 6. Refinement

Hydrogen atoms were included in calculated positions and treated as riding on their parent C atoms with C—H = 0.95Å and *U*_iso_(H) = 1.2*U*_eq_(C). The maximum and minimum residual electron density peaks of 2.15 and 1.73 eÅ^-3^, respectively, were located 0.93 Å and 1.00 Å from the Hg atom.

### Crystal data

**Table:**

[HgCl_2_(C_20_H_12_N_2_)]	*Z* = 2
*M**_r_* = 551.81	*F*(000) = 520
Monoclinic, *P*2/*c*	*D*_x_ = 2.035 Mg m^−^^3^
*a* = 12.285 (3) Å	Mo *K*α radiation, λ = 0.71073 Å
*b* = 4.8482 (10) Å	µ = 8.85 mm^−^^1^
*c* = 15.271 (3) Å	*T* = 173 K
β = 98.00 (3)°	Block, colourless
*V* = 900.7 (3) Å^3^	0.18 × 0.16 × 0.16 mm

### Data collection

**Table:**

Bruker SMART 1000 CCD area-detector diffractometer	1585 independent reflections
Radiation source: fine-focus sealed tube	1512 reflections with *I* > 2σ(*I*)
Graphite monochromator	*R*_int_ = 0.033
ω and phi scan	θ_max_ = 25.0°, θ_min_ = 2.7°
Absorption correction: multi-scan (*SADABS*; Sheldrick, 1995)	*h* = −11→14
*T*_min_ = 0.222, *T*_max_ = 0.243	*k* = −5→5
4238 measured reflections	*l* = −18→17

### Refinement

**Table:**

Refinement on *F*^2^	Primary atom site location: structure-invariant direct methods
Least-squares matrix: full	Secondary atom site location: difference Fourier map
*R*[*F*^2^ > 2σ(*F*^2^)] = 0.028	Hydrogen site location: inferred from neighbouring sites
*wR*(*F*^2^) = 0.070	H-atom parameters constrained
*S* = 0.92	*w* = 1/[σ^2^(*F*_o_^2^) + (0.0394*P*)^2^ + 2.093*P*] where *P* = (*F*_o_^2^ + 2*F*_c_^2^)/3
1585 reflections	(Δ/σ)_max_ < 0.001
114 parameters	Δρ_max_ = 2.15 e Å^−^^3^
0 restraints	Δρ_min_ = −1.73 e Å^−^^3^

### Special details

**Table:**

Geometry. All e.s.d.'s (except the e.s.d. in the dihedral angle between two l.s. planes) are estimated using the full covariance matrix. The cell e.s.d.'s are taken into account individually in the estimation of e.s.d.'s in distances, angles and torsion angles; correlations between e.s.d.'s in cell parameters are only used when they are defined by crystal symmetry. An approximate (isotropic) treatment of cell e.s.d.'s is used for estimating e.s.d.'s involving l.s. planes.
Refinement. Refinement of *F*^2^ against ALL reflections. The weighted *R*-factor *wR* and goodness of fit *S* are based on *F*^2^, conventional *R*-factors *R* are based on *F*, with *F* set to zero for negative *F*^2^. The threshold expression of *F*^2^ > σ(*F*^2^) is used only for calculating *R*-factors(gt) *etc*. and is not relevant to the choice of reflections for refinement. *R*-factors based on *F*^2^ are statistically about twice as large as those based on *F*, and *R*- factors based on ALL data will be even larger.

### Fractional atomic coordinates and isotropic or equivalent isotropic displacement parameters (Å^2^)

**Table:**

	*x*	*y*	*z*	*U*_iso_*/*U*_eq_	
Hg1	0.5000	−0.01536 (4)	0.2500	0.01426 (13)	
Cl1	0.61049 (9)	−0.1178 (2)	0.38614 (6)	0.0215 (3)	
C6	0.1775 (4)	0.9118 (11)	0.3998 (3)	0.0188 (9)	
C5	0.3948 (4)	0.3872 (9)	0.3923 (3)	0.0178 (9)	
H5	0.4536	0.3071	0.4310	0.021*	
C3	0.2446 (3)	0.7051 (9)	0.3688 (3)	0.0161 (9)	
C2	0.2249 (4)	0.6136 (9)	0.2821 (3)	0.0193 (9)	
H2	0.1656	0.6871	0.2425	0.023*	
C7	0.1230 (4)	1.0887 (10)	0.4276 (3)	0.0179 (9)	
C8	0.0599 (3)	1.2960 (8)	0.4641 (3)	0.0156 (8)	
C4	0.3310 (4)	0.5860 (11)	0.4251 (3)	0.0196 (9)	
H4	0.3457	0.6411	0.4853	0.024*	
C1	0.2929 (4)	0.4134 (11)	0.2537 (3)	0.0197 (9)	
H1	0.2795	0.3527	0.1940	0.024*	
C10	0.1000 (4)	1.4112 (11)	0.5461 (3)	0.0205 (9)	
H10	0.1682	1.3511	0.5772	0.025*	
C9	−0.0398 (4)	1.3864 (10)	0.4180 (3)	0.0197 (9)	
H9	−0.0665	1.3092	0.3619	0.024*	
N1	0.3772 (3)	0.3030 (7)	0.3080 (2)	0.0141 (7)	

### Atomic displacement parameters (Å^2^)

**Table:**

	*U*^11^	*U*^22^	*U*^33^	*U*^12^	*U*^13^	*U*^23^
Hg1	0.01265 (19)	0.01679 (18)	0.01374 (17)	0.000	0.00326 (11)	0.000
Cl1	0.0189 (6)	0.0284 (7)	0.0167 (5)	0.0057 (4)	0.0013 (4)	0.0040 (4)
C6	0.018 (3)	0.019 (2)	0.020 (2)	−0.001 (2)	0.0039 (19)	0.0006 (19)
C5	0.014 (2)	0.020 (2)	0.018 (2)	0.0032 (18)	0.0009 (17)	−0.0011 (17)
C3	0.016 (2)	0.015 (2)	0.018 (2)	−0.0011 (17)	0.0063 (16)	−0.0011 (16)
C2	0.018 (2)	0.020 (3)	0.019 (2)	0.0059 (19)	0.0012 (18)	0.0001 (18)
C7	0.018 (3)	0.020 (2)	0.016 (2)	−0.002 (2)	0.0014 (18)	0.0005 (19)
C8	0.017 (2)	0.015 (2)	0.0162 (19)	−0.0015 (17)	0.0068 (16)	0.0019 (16)
C4	0.018 (3)	0.023 (2)	0.018 (2)	0.001 (2)	0.0053 (18)	−0.005 (2)
C1	0.020 (3)	0.024 (2)	0.017 (2)	0.002 (2)	0.0060 (19)	−0.0011 (19)
C10	0.019 (3)	0.022 (2)	0.020 (2)	0.004 (2)	0.0039 (19)	0.002 (2)
C9	0.020 (2)	0.021 (2)	0.018 (2)	0.0003 (19)	0.0041 (18)	−0.0036 (18)
N1	0.0115 (18)	0.0172 (18)	0.0145 (16)	−0.0012 (14)	0.0053 (13)	−0.0004 (14)

### Geometric parameters (Å, º)

**Table:**

Hg1—Cl1^i^	2.3719 (12)	C2—H2	0.9500
Hg1—Cl1	2.3719 (12)	C7—C8	1.428 (6)
Hg1—N1^i^	2.412 (3)	C8—C9	1.396 (6)
Hg1—N1	2.412 (3)	C8—C10	1.397 (6)
C6—C7	1.202 (8)	C4—H4	0.9500
C6—C3	1.420 (6)	C1—N1	1.344 (6)
C5—N1	1.339 (5)	C1—H1	0.9500
C5—C4	1.380 (7)	C10—C9^ii^	1.386 (7)
C5—H5	0.9500	C10—H10	0.9500
C3—C2	1.385 (6)	C9—C10^ii^	1.386 (7)
C3—C4	1.395 (6)	C9—H9	0.9500
C2—C1	1.389 (7)		
			
Cl1^i^—Hg1—Cl1	155.82 (6)	C9—C8—C7	120.7 (4)
Cl1^i^—Hg1—N1^i^	97.08 (8)	C10—C8—C7	119.2 (4)
Cl1—Hg1—N1^i^	98.33 (8)	C5—C4—C3	119.1 (4)
Cl1^i^—Hg1—N1	98.33 (8)	C5—C4—H4	120.4
Cl1—Hg1—N1	97.08 (8)	C3—C4—H4	120.4
N1^i^—Hg1—N1	100.42 (16)	N1—C1—C2	122.1 (4)
C7—C6—C3	178.3 (5)	N1—C1—H1	119.0
N1—C5—C4	122.5 (4)	C2—C1—H1	119.0
N1—C5—H5	118.7	C9^ii^—C10—C8	119.8 (4)
C4—C5—H5	118.7	C9^ii^—C10—H10	120.1
C2—C3—C4	118.3 (4)	C8—C10—H10	120.1
C2—C3—C6	120.8 (4)	C10^ii^—C9—C8	120.1 (4)
C4—C3—C6	120.9 (4)	C10^ii^—C9—H9	120.0
C3—C2—C1	119.3 (4)	C8—C9—H9	120.0
C3—C2—H2	120.3	C5—N1—C1	118.6 (4)
C1—C2—H2	120.3	C5—N1—Hg1	121.5 (3)
C6—C7—C8	177.8 (5)	C1—N1—Hg1	119.7 (3)
C9—C8—C10	120.1 (4)		
			
C4—C3—C2—C1	−1.6 (7)	C4—C5—N1—C1	−1.0 (7)
C6—C3—C2—C1	179.3 (5)	C4—C5—N1—Hg1	174.6 (4)
N1—C5—C4—C3	−0.1 (8)	C2—C1—N1—C5	0.8 (7)
C2—C3—C4—C5	1.4 (7)	C2—C1—N1—Hg1	−174.9 (4)
C6—C3—C4—C5	−179.5 (5)	Cl1^i^—Hg1—N1—C5	168.6 (3)
C3—C2—C1—N1	0.6 (8)	Cl1—Hg1—N1—C5	7.3 (3)
C9—C8—C10—C9^ii^	0.7 (8)	N1^i^—Hg1—N1—C5	−92.5 (3)
C7—C8—C10—C9^ii^	179.7 (5)	Cl1^i^—Hg1—N1—C1	−15.8 (3)
C10—C8—C9—C10^ii^	−0.7 (8)	Cl1—Hg1—N1—C1	−177.1 (3)
C7—C8—C9—C10^ii^	−179.7 (5)	N1^i^—Hg1—N1—C1	83.0 (3)

Symmetry codes: (i) −*x*+1, *y*, −*z*+1/2; (ii) −*x*, −*y*+3, −*z*+1.
